# Rabies virus as vector for development of vaccine: pros and cons

**DOI:** 10.3389/fvets.2024.1475431

**Published:** 2024-09-25

**Authors:** Yan Li, Houcheng Zhou, Qian Li, Xiaoxiao Duan, Fuxiao Liu

**Affiliations:** ^1^College of Veterinary Medicine, Qingdao Agricultural University, Qingdao, China; ^2^Qingdao Center for Animal Disease Control and Prevention, Qingdao, China; ^3^Laizhou Zhenzhu Station for Animal Husbandry and Veterinary Medicine, Yantai, China

**Keywords:** rabies virus, reverse genetics, live-attenuated RV-vectored vaccine, inactivated RV-vectored vaccine, replication-deficient RV-vectored vaccine

## Introduction

Rabies is a zoonotic viral disease that causes encephalitis in humans and other mammals, such as dogs, bats, raccoons, and foxes (1). This disease generally includes two clinical forms, namely furious rabies and paralytic rabies (2). The former is characterized by hyperactivity and hallucinations; the latter is characterized by paralysis and coma. Rabies induces progressive and fatal inflammation of the brain and spinal cord. Once its clinical sign begins, the mortality rate is virtually 100% in humans. Rabies is still a serious public problem in over 150 countries and territories, mainly in Asia and Africa (3).

The etiological agent of rabies is rabies virus (RV), a typical neurotropic virus. Its transmission is commonly through saliva, bites, scratches, or direct contact with mucosa (4). According to the latest taxonomic classification, RV has been renamed lyssavirus rabies, classified into the genus *Lyssavirus* in the family *Rhabdoviridae*. The virion is a bullet-shaped particle with envelope, containing a single-stranded, negative-sense RNA genome, ~11.9 nt in length, encoding five proteins in order: nucleoprotein (N), phosphoprotein (P), matrix protein (M), glycoprotein (G), and RNA-dependent RNA polymerase (RdRp or L protein).

The RV genome can be readily modified to accommodate a foreign antigen sequence for rescuing a recombinant virus using revere genetics (5). The recombinant, if demonstrated to be able to induce specific immune responses *in vivo*, would play a potential role in developing the RV-vectored vaccine. To date, there have been three types of RV-vectored vaccines: live-attenuated, inactivated and replication-deficient patterns. They showed their individual strengths and weaknesses, both of which were critically discussed here.

## Reverse genetics for recovery of antigen-expressing RV

The reverse genetics platform of RV was initially reported in 1994 (6), subsequently revolutionizing the researches on RV and even on other RNA viruses. This reverse genetics platform includes four plasmids, and one cell line. The four-plasmid system contains one viral cDNA clone, and three helper plasmids that separately express N, P and L proteins. An RV-vectored cDNA clone generally possesses an extra transcriptional unit of foreign antigen. Recovery of RV involves co-transfection of these four plasmids into a cell line [for a review on the protocol, see (5)]. The rescued chimeric RV will be characterized for verifying its ability in the expression of foreign antigen *in vitro*, and then subjected to the animal test for unveiling its potential in eliciting specific immune responses. Table 1 exhibits a range of RV-vectored candidate vaccines against distinct pathogens.

**Table 1:** Rabies virus-vectored candidate vaccines against distinct pathogens.

**Targeted pathogen**	**Rabies virus-expressed antigen**	**Type of vaccine**	**Animal test**	**Animal**	**References**
*Bacillus anthracis*	Protective antigen D-4	Inactivated	Yes	Mice	(7)
*Borrelia burgdorferi*	BBI39	Inactivated	Yes	Mice	(8)
BEFV	Glycoprotein	Live	Yes	Mice	(9)
CDV	H protein	Live	Yes	Dogs	(10)
Canine parvovirus	Virion protein 2	Inactivated and live	Yes	Mice	(11)
Ebola virus	Glycoprotein	Inactivated	Yes	Mice and dogs	(12)
*E. granulosus*	EG95	Live	Yes	Mice	(13)
EV, SV, MV, or (and) LV	Glycoprotein or GPC	Inactivated	Yes	*Macaca fascicularis*	(14)
FHV-1	Glycoprotein B	Inactivated	Yes	Mice and cats	(15)
Hendra virus	G protein	Live	Yes	Mice	(16)
HIV-1	Gag	Replication-deficient	Yes	Mice	(17)
HIV-1	gp160	Live	Yes	Mice	(18, 19)
Lassa virus	Glycoprotein	Inactivated	Yes	Mice and guinea pigs	(20)
LCMV	Glycoprotein	Replication-deficient	Yes	Mice	(21)
Marburg virus	Glycoprotein	Inactivated and live	Yes	Mice	(22, 23)
MERS-CoV	S1 subunit	Inactivated	Yes	Mice, camels and alpacas	(24)
MERS-CoV	Infused S1 subunit	Replication-deficient	Yes	Mice	(25)
Nipah virus	A.G. or F.G.	Live	Yes	Mice and pigs	(26)
Nipah virus	G protein	Inactivated and live	Yes	Mice	(27)
PPRV	H or F protein	N.A.	N.A.	N.A.	(28)
SARS-CoV-2	S1 subunit	Inactivated and live	Yes	Hamsters and mice	(29–32)
SARS-CoV-2	TRBDT of S1 subunit	Inactivated	Yes	Mice	(33)
SARS-CoV-2	RBD of SARS-CoV-2	Inactivated	Yes	Cats and dogs	(34)
SFTSV	Gn	Live	Yes	Mice	(35)
SHIV and SIV	SHIV_89.6P_ Env and SIV_mac239_ Gag	Live	Yes	Rhesus macaques	(36)
RVFV	Gn ectodomain	Inactivated	Yes	Mice	(37)

A.G. or F.G., attachment glycoprotein or fusion glycoprotein; BEFV, bovine ephemeral fever virus; CDV, canine distemper virus; *E. granulosus, Echinococcus granulosus*; EV, SV, MV or (and) LV, Ebola virus, Sudan virus, Marburg virus or (and) Lassa virus; FHV-1, feline herpesvirus-1; GPC, glycoprotein complex; gp160, glycoprotein 160; HIV-1, human immunodeficiency virus type 1; LCMV, lymphocytic choriomeningitis virus; MERS-CoV, Middle East respiratory syndrome coronavirus; N.A., not available; PPRV, peste des petits ruminants virus; RBD, receptor-binding domain; RVFV, Rift Valley fever virus; SARS-CoV-2, severe acute respiratory syndrome coronavirus type 2; SFTSV, severe fever with thrombocytopenia syndrome virus; SHIV, simian-human immunodeficiency virus; SIV, simian immunodeficiency virus; TRBDT, tandem receptor-binding domain trimer.

## Live-attenuated RV-vectored vaccine

The requirements for developing live-attenuated RV-vectored vaccines (LRVs) include many basic features: no or low virulence in hosts, ability of reaching high-level titers *via* cell culture, continuously eliciting cellular and humoral immune responses, thermal stability, and last but not least, genetic stability of foreign sequence. LRVs are factually the replication-competent RVs, able to replicate continuously in animals. They usually require fewer doses, and moreover provide more long-lasting protection than inactivated vaccines. A single-dose vaccination schedule is enough to induce high titers of specific antibodies *in vivo* (27). Moreover, LRVs offer more comprehensive and long-lasting protection in animals from pathogens.

Besides the humoral immunity, the LRVs also induce cellular immunity, because viable RVs propagate in host's cells and express endogenous antigens for further processing and presentation. The cell-mediated immune response is crucial for eliminating pathogens inside the host's cells. Zheng et al. (9) evaluated T-cell-mediated immune responses in mice elicited by an LRV that expressed the glycoprotein of bovine ephemeral fever virus (BEFV). Both RV- and BEFV-specific cytokines, interferon-γ and interleukin-4, could be identified to secrete in lymphocytes. This LRV induced more robust T helper 1 (Th1) and Th2 cell-mediated immunities than the parent RV *via* the single-dose immunization strategy (9). However, not all LRVs can induce the cellular immunity. McKenna et al. (36) previously demonstrated the immunogenicity of LRV expressing simian-human immunodeficiency virus (SHIV)_89.6P_ Env in rhesus macaques. Humoral immunity against RV G protein and SHIV_89.6P_ Env was detectable after the initial immunization, whereas the cell-mediated immune response was not identified against the SHIV antigens (36).

Although LRVs have been widely demonstrated to be powerful in eliciting protective immune responses in animals, there are still two weaknesses that should not be neglected for the development of LRVs. The first one is the potential risk in reversion to virulence. Most RNA viruses are genetically unstable during genomic replication, due to the low-fidelity characteristics of their RdRps (38). RVs have been rapidly evolving (39), and been even recombining with one another (40). A single mutation of amino acid in its G protein will quicken its spread, and even intensify its pathogenicity (41, 42). Therefore, the potential reversion to virulence hampers the further application of LRVs. The other weakness, albeit rarely reported as yet, should not also be neglected to design an LRV. This weakness is that a foreign sequence is possibly unstable in a chimeric RV genome, since the foreign sequence is theoretically uninvolved in virus-associated events. If a certain foreign sequence is deleted from a chimeric RV genome, the resultant RV would be still a replication-competent strain, but lose its own primary properties of vector vaccine.

## Inactivated RV-vectored vaccine

The inactivated RV-vectored vaccine (IRV) is produced as a killed version of antigen-expressing RV. The prerequisite for developing a certain IRV is that a target antigen must be incorporated into the envelope of chimeric RV virion (43). Some viral glycoproteins, if expressed through the RV vector in cells, can be further processed, modified and finally transported to the cell surface. These viral glycoproteins, as membrane-spanning proteins, will be embedded into the cell envelope. Along with the viral budding, the foreign antigen can be incorporated into the envelope of RV virion. Many viral glycoproteins, like those of Lassa virus (20) and Ebola virus (12), were reported to have such a feature, therefore playing a potential role in the development of the IRVs.

However, not all viral glycoproteins can be directly used as a complete foreign antigen for preparing the IRV. Some ones need to be modified for the incorporation into RV virions. For instance, Rift Valley fever virus (RVFV) morphogenesis is by means of budding from the Golgi complex. In other words, the RVFV glycoprotein is unable to be transported to the cell membrane, therefore requiring the replacement of its trans-membrane domain and cytoplasmic tail with those of RV G protein for the incorporation of a fusion protein into the RV virion (37). Such a fusion modification was also reported to be used in non-viral proteins for preparing the IRV (7).

Compared with that of LRV, the most significant advantage of IRV is its good safety profile in animals, due to the recombinant RV functioning as an inactivated virion. Neither RV mutation nor virulence reversion can occur in IRV-inoculated animals. Although the recombinant RV is chemically inactivated, its immunogenicity can be even completely retained. The inactivated recombinant RV induces not only its own immune responses, but also more importantly, specific neutralizing antibodies against the target pathogen. One research group recently constructed an IRV, named CORAVAX™, against severe acute respiratory syndrome coronavirus-2 (SARS-CoV-2) (32). A single dose of CORAVAX™ vaccine was demonstrated not only to elicit the high-level SARS-CoV-2-specific antibodies, but also to prevent weight loss, viral loads, lung inflammation, and cytokine storm in hamsters (29). Subsequently using the mouse models, this group further screened adjuvants for the maximum level of antibody titers, negated the concerns about pre-existing RV-vectored immunity, and determined its potential as a long-lasting IRV against SARS-CoV-2 (31). Extra experiments should be conducted to demonstrate whether the candidate vaccine CORAVAX™ is also effective in nonhuman primates.

Although the IRVs show a great potential in clinical use, there are still a few disadvantages to them. The most representative one is that they are generally less effective than their live counterparts. An LRV induces the immune response so robust that a single dose is enough to the immunization of animals, whereas an IRV commonly requires booster injections (23, 27). The immunity effect is involved in a dose-dependent manner for IRV-inoculated animals. The prime-boost immunization strategy is inconvenient, because at least two rounds of injection are needed. Another non-negligible issue is the potential risk of incomplete inactivation during IRV production. Such a risk can be excluded to the maximum extent through use of more reliable inactivants following a standard procedure of virulence inactivation.

## Replication-deficient RV-vectored vaccine

Replication-deficient viruses are functionally defective in genome replication or (and) virion assembly (44). Construction of replication-deficient RV was reported as early as 1995 (45). A replication-deficient RV is a pseudo-live virion, which is able to infect a cell, but completes only one single-cycle replication, and more importantly cannot produce the replication-competent progenies. The replication-deficient RV-vectored vaccine (RRV) provides a new form of RV-vectored vaccine that combines many advantages of LRV and IRV, such as good safety and robust immunogenicity (Table 1).

It has been showed that the M gene-deleting RV confers 4-fold higher titers of neutralizing antibodies than does a commercially available vaccine in monkeys within 10 days after vaccination (46). The RV P protein is a multifunctional protein, required not only for RV replication, but also for innate immunity evasion (47). Takayama-Ito et al. (21) constructed a P gene-deficient RV that expressed the glycoprotein precursor of lymphocytic choriomeningitis virus (LCMV). Such a replication-deficient RV was subsequently proven to be a promising RRV candidate, characterized by dual immunities against LCMV and RV (21).

The G gene can be removed from a chimeric RV genome for the development of RRV. Gomme et al. (17) constructed a replication-deficient RV through deleting the G gene from an RV vector that expressed the HIV-1 Gag. This RRV was demonstrated to induce weaker RV-specific antibody responses, but equivalent HIV-1 Gag-specific CD8^+^ T cell responses. These responses could be considerably enhanced through boosting with the G gene-deleting RV complemented with one heterologous glycoprotein (17). Thus, the labor-consuming prime-boost strategy may be necessary for the RRV-based immunization. The G gene-deficient RV is also named single-cycle virus, which is still capable of budding from the cell membrane, but shows a 30-fold lower efficiency (48). The budded virions are able neither of attachment nor of entry into secondary host cells (49). Therefore, another drawback to RRVs is the difficulty in obtaining a high-titer viral stock through cell culture.

## Conclusions

RV is an attractive candidate for designing and producing virus-vectored vaccines. This virus allows for foreign antigen expression, and even incorporation into the mature virions. A large number of reports, concerning LRVs, IRVs, and RRVs (Table 1), have highlighted their immune efficacies, antigen-delivering abilities, and safety profiles in animals. Nevertheless, a few issues should not be neglected regarding their production and application. Firstly, although the LRVs are functionally robust in eliciting both humoral and cellular immunities, all safety risks must be eliminated before they can be used. Secondly, manufacturers should pay attention to some accidents, caused by the incomplete inactivation of viruses. Last but not least, because the RV G protein is highly immunogenic in animals, the immune response to it may interfere with responses an RV-vectored vaccine confers to foreign proteins (43).
